# Habitat-Mediated Dive Behavior in Free-Ranging Grey Seals

**DOI:** 10.1371/journal.pone.0063720

**Published:** 2013-05-07

**Authors:** Mark Jessopp, Michelle Cronin, Tom Hart

**Affiliations:** 1 Coastal & Marine Research Centre, Environmental Research Institute, University College Cork, Cork, Ireland; 2 Department of Zoology, University of Oxford, Oxford, United Kingdom; Aristotle University of Thessaloniki, Greece

## Abstract

Understanding the links between foraging behaviour and habitat use of key species is essential to addressing fundamental questions about trophic interactions and ecosystem functioning. Eight female grey seals (*Halichoerus grypus*) were equipped with time-depth recorders linked to Fastloc GPS tags following the annual moult in southwest Ireland. Individual dives were coupled with environmental correlates to investigate the habitat use and dive behaviour of free-ranging seals. Dives were characterised as either pelagic, benthic, or shallow (where errors in location and charted water depth made differentiating between pelagic and benthic dives unreliable). Sixty-nine percent of dives occurring in water >50 m were benthic. Pelagic dives were more common at night than during the day. Seals performed more pelagic dives over fine sediments (mud/sand), and more benthic dives when foraging over more three-dimensionally complex rock substrates. We used Markov chain analysis to determine the probability of transiting between dive states. A low probability of repeat pelagic dives suggests that pelagic prey were encountered *en route* to the seabed. This approach could be applied to make more accurate predictions of habitat use in data-poor areas, and investigate contentious issues such as resource overlap and competition between top predators and fisheries, essential for the effective conservation of these key marine species.

## Introduction

Animal behaviour is best interpreted in the context of its local environment. The third dimension of water depth is therefore fundamental to the study of behaviour and ecology in marine systems [1]. However, studies of marine predator behaviour are limited by the fact that it is practically impossible to directly observe individuals and classify behaviour. Biotelemetry has emerged as one of the most successful methods to study free-living animals, with technological innovation in archival tags enabling us to infer behaviours from measurable parameters such as location, dive profiles, heart rate, and orientation [2].

A number of previous studies have investigated associations between broad-scale habitat features such as depth, temperature and ecoregions, and diving marine mammals [3], [4], [5], [6]. However, to understand how animals use the environment, we need to understand not just the spatio-temporal distribution of animals, but the proximate causes of change in distribution. Behavioural responses to change in the environment give us an understanding of what features are important. However, linking behaviour with habitat is often impeded due to uncertainties in position estimates on a scale suitable to infer fine-scale habitat use [1]. Systems such as the ARGOS satellite system suffer from large inaccuracies in position estimates [7], with error on position estimates often exceeding the spatial scale at which the associated environmental variables are measured. Standard GPS receivers typically require over 30 seconds to obtain a position fix [8], making them unsuitable for use on diving animals that spend little time at the surface. The recent advent of fastloc GPS technology, which requires less than half a second at the surface to obtain accurate position fixes, means that we can now obtain accurate positional data in diving marine animals [9], [10]. Fine-scale animal locations associated with environmental data now allow us to be much more confident about the habitat encountered by diving species. This also allows us to identify when animals transit habitats, and infer changes in behaviour.

Grey seals (*Halichoerus grypus*) are generalist feeders, opportunistically consuming a wide range of prey species [11], [12], [13]. They are generally coastal foragers [14], although they can range widely [15], enabling seals to forage over many different habitats. As demersal feeders [14], grey seals are an excellent model species to investigate the relationship between dive behaviour, prey consumption, and seafloor habitat. Multivariate analysis has shown fish communities to be correlated with depth, latitude and seabed type on the continental shelf [16], while reef and demersal fish species have both been shown to correlate with specific habitat variables including sediment type and structural complexity [17], [18]. In UK and Irish waters, extensive spatial and temporal variation in gadiform, perciform and flatfish consumption has been noted in grey seal diet, likely due to variation in habitat-mediated prey availability [19]. Determining these relationships may also help to address conservation issues such as the perceived conflict between seals and commercial fisheries in terms of spatial overlap and competition for resources [20].

Efforts to characterise the seabed, largely for territorial claims and exploitation of mineral resources, means that increasingly, fine-scale sediment data are available. This can now be combined with spatially accurate dive data to investigate dive behaviour in relation to habitat in free-ranging grey seals. We present new analyses to characterise dive behaviour, identifying where switching between dive behaviours occurs, and investigate how this may relate to habitat use in free-ranging grey seals.

Our aims are:

1. To identify different types of dive behaviour, in particular, benthic versus pelagic diving.
2. To investigate correlates between dive types and sediment type, i.e. whether feeding occurs more on the benthos over certain sediments.
3. To investigate diurnal differences in dive behaviour and habitat use.

## Methods

### Ethics statement

All grey seal handling and tagging procedures were reviewed and approved by the National Parks and Wildlife Service, and carried out under licence Number C35/2008 issued by National Parks and Wildlife Service, Department of Arts, Heritage and the Gaeltacht. Seals were anesthetized during the tagging process and handling times were minimized to reduce animal stress.

Capture of grey seals and deployment of Fastloc/GSM tags was carried out at a haul-out site in southwest Ireland on the Trá Ban, Great Blasket Island, County Kerry. Tagging was conducted in February 2009 to coincide with the completion of the female moult (Pers. Obs). Researchers approached the haul-out site by sea using motorized, rigid inflatable boats, and captured individuals in hoop nets on the shore. Seals were weighed to the nearest 1 kg and anaesthetised using 0.5 mg of Zoletil (© Virbac, a combination of a dissociative anesthetic agent, tiletamine hypochloride, and a tranquilizer, zolazepam hypochloride) per kg, delivered intravenously. Curvilinear length (nose to end of tail) and girth (immediately posterior to the fore-flippers) of each animal were measured to the nearest cm. The fur was dried with paper towels and degreased using acetone prior to securing a GPS/GSM tag (Sea Mammal Research Unit St Andrews University, Scotland) to the fur at the base of the skull using 2-part quick-setting epoxy adhesive (RS components).

The tag (10×7×4 cm, 370 g, full specifications available at http://www.smru.st-andrews.ac.uk/protected/downloads/GPS_Phone_Tag22.pdf) incorporates a hybrid GPS system Fastloc (Wildtrack Telemetry Systems, Leeds, UK) capturing GPS pseudo-range data that are compressed into 30 byte records and post-processed with archived orbitography data to calculate location. The significant advantage of this system is that the required data capture requires less than half a second at the surface, enabling frequent and accurate positions being acquired at sea (up to 26 m accuracy, depending on number of satellites available [10]). The tags were programmed to attempt a location fix every 30 minutes but were only successful if the fix attempt coincided with the animal being at the surface.

Dives were defined as beginning when the tag was below 1.5 m for 8 seconds and ended when the tag returned to a depth shallower than 1.5 m. This excludes periods of rest and travelling at sea from analysis of dive events. Location for dives not occurring at precisely the time of position fixes were derived using straight-line interpolation between position fixes so that water depth and habitat variables could be associated. Location and dive data were stored onboard the tag and sent ashore via a data link call when the seal came within range of the coastal GSM mobile phone network. The tags summarised dive events to maximum depth, duration, and depth at each 1/10^th^ of dive duration to minimise data volume for transmission. Only maximum depth and duration were used for this analysis.

Only location fixes that used five or more satellites to determine position were used for analysis. The additional error (to GPS accuracy) associated with interpolated locations (those occurring between position fixes) was calculated as the potential travel distance based on time between fixes and the maximum recorded swimming speed, minus the actual distance between fixes and scaled by the number of dives occurring between fixes:
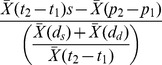
where 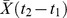 is the mean difference in time (seconds), 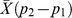 is the mean distance between fixes (m), and *s* is the maximum swimming speed recorded between successive position fixes. 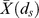 is the mean surface duration, 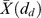 is the mean dive duration.

**Figure 1:**
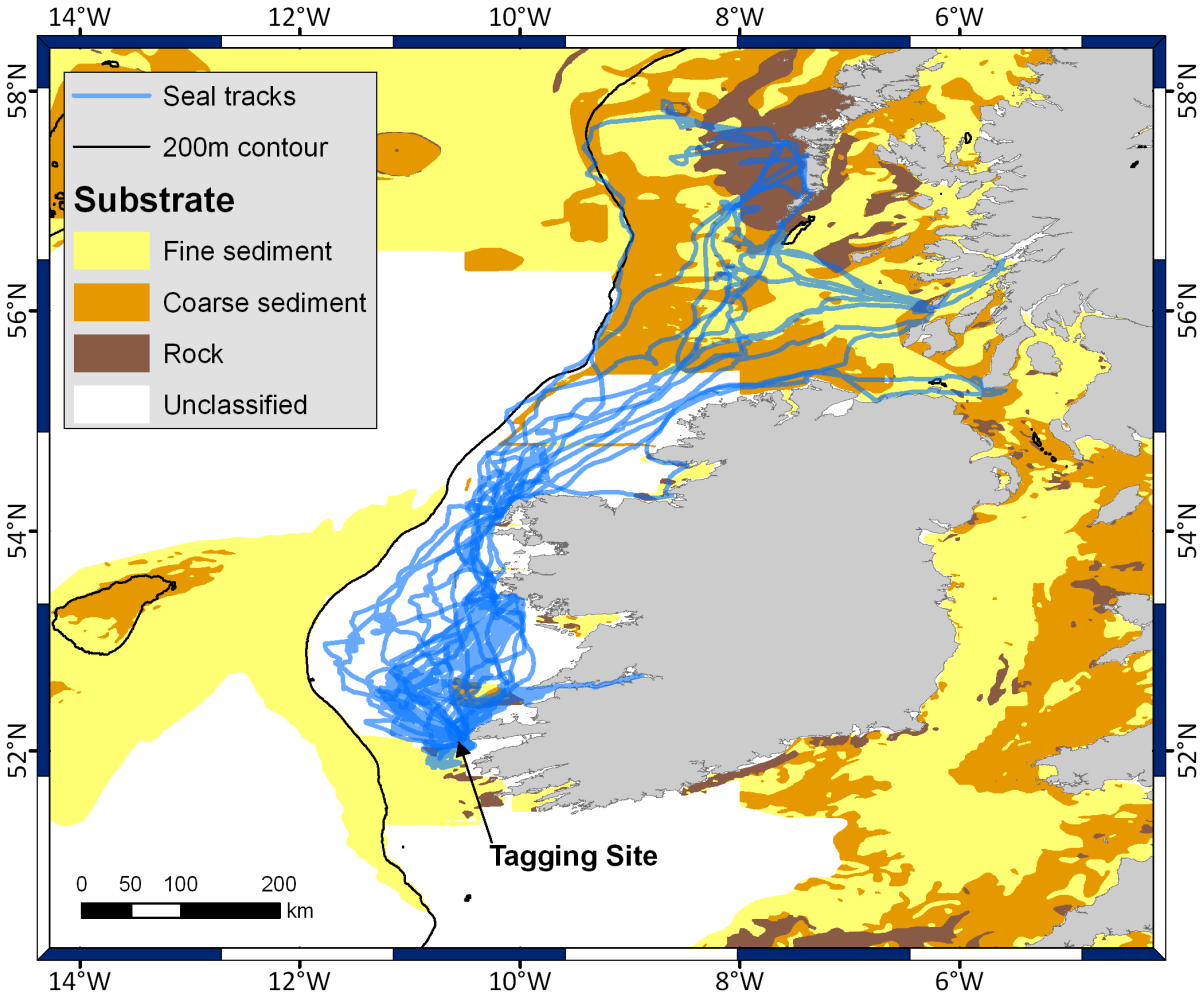
Location of tagging site in Southwest Ireland, with seal tracks and seabed sediment type. Seals (n = 8) were tagged with a Fastloc GPS/GSM tag, and foraged over a range of different sediment types, from fine mud and sand through to more three-dimensionally complex rock substrates.

We used Spatial Analyst to extract values for bathymetry and sediment type to each dive in ArcMap 10 (ESRI). The GEBCO_08 global 30 arc-second grid altimeter dataset for ocean bathymetry, freely available through the British Oceanographic Data Centre (BODC), was used to determine water depth. Sediment type follows the EUNIS 2007–11 classification system and is based on the predictive EUNIS seabed habitat map for the North Sea and Celtic Sea, created using pre-processed input datasets for substrate, biological zone and energy using raster input layers with a cell size of 0.003 decimal degrees (∼167×333 m). These data were downloaded from the EUSeaMap web portal administered through the JNCC (http://jncc.defra.gov.uk/page-5040). Sediment types were pooled to create three broad habitat classes for the analysis: fine (mud/sand), coarse (gravel/mixed ground), and rocky (rock/till) sediments. Some areas over which seals foraged have not been surveyed, and were therefore classed as ‘unclassified’ (Fig. 1). Day and night determinations were made based on the timing of local sunrise and sunset at each given dive date, time and location (latitude/longitude).

**Figure 2:**
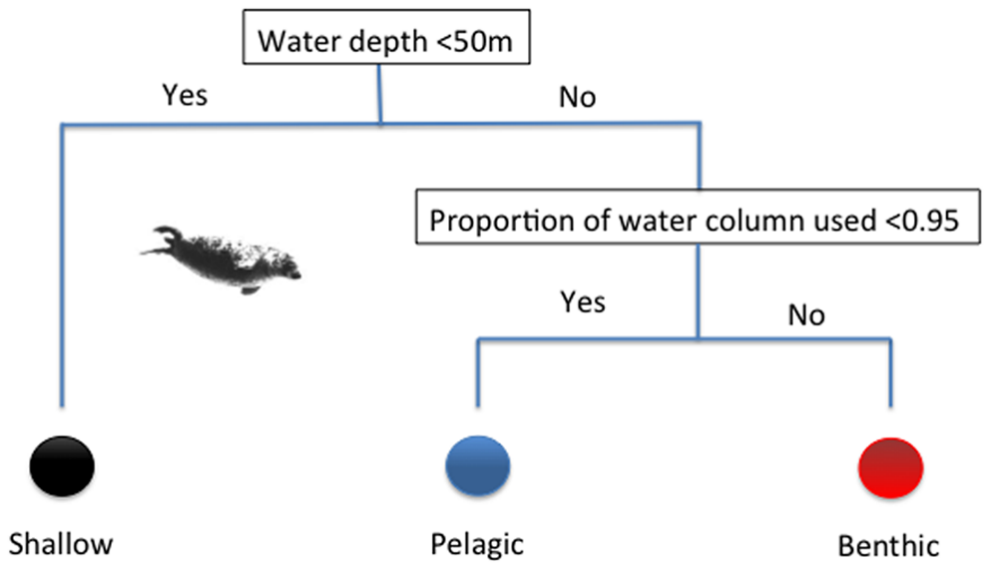
Schematic of dive classification. Dives were identified as ‘shallow’, ‘benthic’, and ‘pelagic’. All dives in water depth of less than 50 m were classified as ‘shallow’. Remaining dives were further divided into ‘benthic’ and ‘pelagic’ dives based on proximity of dive to the seabed.

Dive types were assigned using conditional statements (Fig. 2). Disproportionately high errors in the ratio of water depth to dive depth in water less than 50 m resulted in low confidence in describing dives as *pelagic* or *benthic*. These dives were therefore classified as *shallow*. Dives in water deeper than 50 m were classified according to their proximity to the benthos. Two thresholds were identified based on the distribution of data (see Fig. S1). Proximity to the sea floor was calculated as a ratio of dive depth divided by bathymetric depth, with ratios <0.95 classified *pelagic* dives and >0.95 *benthic* dives.

We used Markov chain analysis to calculate transition matrices to estimate the probability of transiting between sediment types and dive types using the ‘statetable’ function within the ‘msm’ package in R 2.12.2 (R Core Development Team, 2012). Transition matrices were calculated for each seal and averaged across seals (reported as mean ± variance) to avoid non-independence. A transition matrix converts all of the data, accounting for repeated measures, into a metric of dependency; i.e. how many times an animal performs the same action consecutively, using the frequency of each dive type for each individual. However, because we have split the data several ways, eg. night vs day, some pseudoreplication is introduced, which is controlled for by including individual in the analysis.

For each sediment type and time of day, the frequency of pelagic dives was calculated and the effect of sediment type, and time of day on frequency were investigated using a linear model in R 2.12.2.

## Results

Eight female grey seals were captured and tagged on February 24 and 25, 2009. Weights of captured seals ranged from 68.2 kg to 121.2 kg (Table 1). Tags operated for approximately 7–8 months (mean duration = 226 days; maximum = 325). In total, 324,900 dives were recorded, with an average depth of 57±48 m, and a maximum dive depth of 455 m. Data are available on request from the authors.

**Table 1:** Details of instrumented female grey seals.

Sealfig Number	Tag Number	Date of last transmission	Tagging duration/days	Weight/kg
**1**	10957	23/07/2009	149	121.2
**2**	11093	16/10/2009	234	78
**3**	11113	30/07/2009	156	69.8
**4**	11101	02/10/2009	220	68.2
**5**	11108	17/05/2009	79	119.2
**6**	11100	16/01/2010	325	115.2
**7**	11095	07/10/2009	224	110.6
**8**	11015	22/12/2009	302	90.2

Mean residual error for location fixes was 12.3 m with over 95% of residual error in position estimates being less than 20 m. Additional average error associated with interpolated locations was calculated at ±67 m. This is considered to be an overestimate, as it does not take into account the vertical distance travelled while diving to depth between successive satellite location fixes.

**Figure 3:**
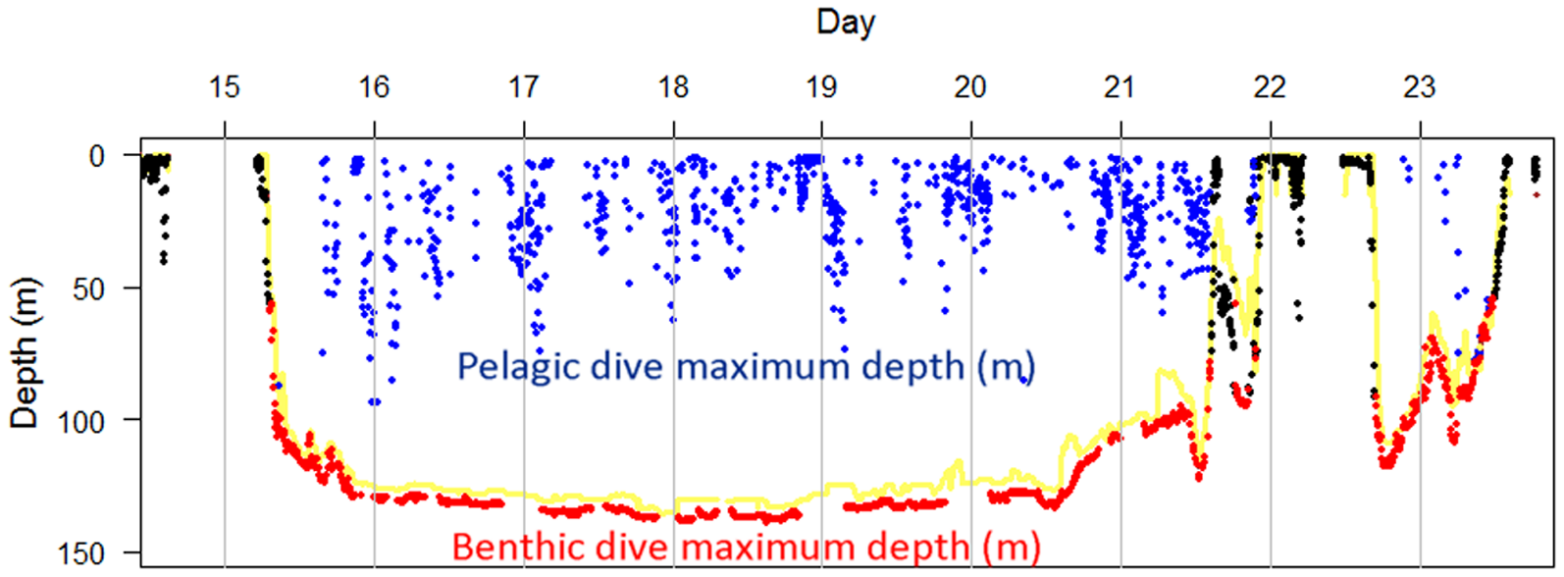
Characteristic dive states of a tagged seal. Maximum dive depths classified according to dive states. Dive states were determined based on water depth and proximity to the benthos. Blue – pelagic dives; red – benthic dives; black – dives in shallow water. Vertical spaces between bathymetry (solid yellow) and individual dives represents the difference between the dive depth and the seabed. Some dives recorded depths greater than the charted bathymetry, but the high correspondence between benthic dives and the bathymetric depth indicates the relatively small error. Error between these two is more likely to be due to error in the bathymetric depth due to differences in tidal height and spatial error. As seals approach shallow waters, this error can result in some dives of greater than 50 m depth being classified as ‘shallow water dives’ when the charted depth at the given location is less than 50 m. Areas where benthic (red) dives approach shallow depths likely indicate approach to haulout locations.

Dives were categorised into one of three types: shallow, benthic, and pelagic. A representative example of a track with classified dive types is given in Fig. 3. All dive types were recorded for all seals, occurring throughout the deployment period. Forty-two percent of all 324,900 dives occurred in water less than 50 m, likely associated with foraging around haulout locations. Of the remaining 189,237 dives occurring in water depths greater than 50 m, 69% were dives to the seabed and 31% were pelagic dives.

Markov chain analysis estimated a high likelihood of a subsequent dive type being the same as the previous dive type (e.g. a benthic dives being followed by another benthic dive). However, a lower probability of repeat pelagic dives (0.139±0.070) was noted when compared to the probability of repeat benthic (0.366±0.110) or shallow (0.393±0.173) dives. The probability of transitions between dive types was <0.05 for all transitions (e.g. shallow to benthic, pelagic to shallow, etc), with an equal probability of benthic dives being followed by pelagic dives and pelagic dives being followed by benthic dives (Table 2). A total of 43,437 dives occurred over mapped sediment types. Sediments ranged from fine sediments such as mud/sand through to more three-dimensionally complex rock (Fig. 1). The probability of successive dives being over the same sediment type was typically<0.07, influenced by the large number of dives occurring over uncharacterised sediment type. This sediment type was included to remove potential bias associated with transitions between two sediment types interspersed by unclassified benthos between them, and to make transparent the effect that missing data would have on transitions. Very low probabilities of transiting between sediment types (all<0.0001) were recorded (Table 3).

**Table 2:** Transition matrix showing the mean (±standard deviation) of the probability of seals transiting from one dive type (shallow, pelagic, benthic) to another.

		Dive number (*i*)		
		Shallow	Pelagic	Benthic
Dive number (*i*+1)	Shallow	**0.393 (±0.173)**	0.001 (±0.000)	0.002 (±0.001)
	Pelagic	0.001 (±0.001)	**0.139 (±0.070)**	0.047 (±0.024)
	Benthic	0.002 (±0.001)	0.047 (±0.024)	**0.366 (±0.110)**

For example, a pelagic dive followed by a benthic dive will occur with a probability of 0.047±0.024. Values in bold along the diagonal represent the probability of dive types being repeated sequentially.

**Table 3:** Transition matrix showing the mean (±standard deviation) of the probability of seals transiting from one habitat type (Fine (mud/sand), Coarse (gravel/mixed), Rock (rock/till)) to another.

		Dive (*i*) sediment			
		Fine	Coarse	Rock	Unclassified
Dive (*i*+1)	Fine	**0.044 (±0.051)**	0.000 (±0.000)	0.000 (±0.000)	0.000 (±0.000)
	Coarse	0.000 (±0.000)	**0.068 (±0.061)**	0.000 (±0.000)	0.000 (±0.000)
	Rock	0.000 (±0.000)	0.000 (±0.000)	**0.064 (±0.052)**	0.001 (±0.000)
	Unclassified	0.001 (±0.000)	0.000 (0.000±)	0.001 (±0.000)	**0.819 (±0.112)**

For example, dives over fine substrate will occur sequentially with a probability of 0.044 (±0.051). Values in bold along the diagonal represent the probability of dives over the same sediment type being repeated sequentially. Unclassified benthic habitats are included to avoid bias that would otherwise be introduced by removal of two sediment types interspersed by unclassified benthos between them.

**Figure 4:**
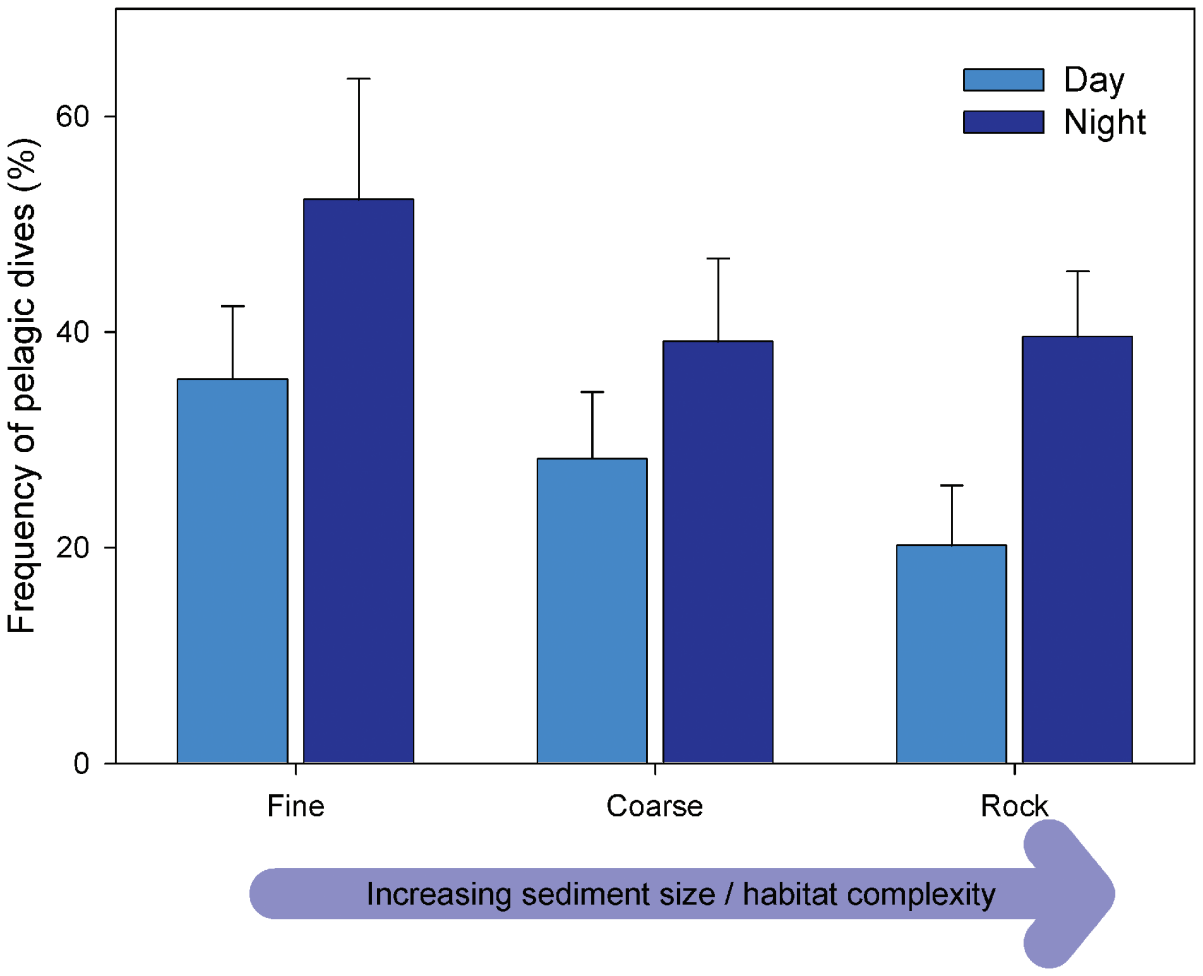
Frequency of pelagic dives occurring over fine, coarse, and rock substrates during day and night. Data represent frequency of pelagic dives out of all dives occurring over areas with available substrate data (n = 43,737), calculated for each seal, and averaged across all (n = 8) seals. For example, over fine sediment, approximately 30% of dives were pelagic during the day, while approximately 50% of dives were pelagic during the night. Error bars represent standard errors. Graph also illustrates how with increasing sediment size/habitat complexity, there is a corresponding decrease in the frequency of pelagic dives.

A linear model was used to investigate the effect of sediment type (where this was known) and time of day on the proportion of pelagic dives. Individual was included to account for non-independence of values from the same seal. The model explained 63% of the total variation (R^2^ = 0.63), and showed that the frequency of pelagic dives varied by sediment type (F_2,34_ = 3.55, P = 0.04), with the frequency of pelagic dives over fine sediment being significantly higher than over both coarse and rock sediments. Overall, pelagic dives were more frequent at night (44%±24%) than day (28%±17%, F_7,34_ = 11.41, P = 0.002), and the frequency of pelagic dives was highly significant between individuals (F_2,34_ = 5.53, P<0.001). The interaction between sediment type and time of day was not significant (F_2,34_ = 0.35, P = 0.710). The frequency of pelagic dives decreased with habitat complexity. Over fine sediment, 36% (±18%) of dives were pelagic during the day, while 52% (±32%) were pelagic during the night, while this was 28% (±17%) by day and 39% (±22%) by night for coarse sediments, and 20% (±16%) by day and 39% (±17%) by night for rock substrates (Fig. 4).

## Discussion

Grey seals primarily made more benthic dives (69%) than pelagic dives (31%), consistent with demersal foraging observed in grey seals elsewhere [14], [19]. Pelagic dives have been previously reported in harbour seals [21], but to our knowledge, have not been reported for grey seals. Pelagic dives likely occurred in other studies on grey seals, but since the authors have not linked dives to bathymetry (likely due to lack of positional accuracy), this behaviour has remained unreported. This study reveals the extent of pelagic diving (31% of dives) and its likely contribution to foraging prey encounters.

The prevalence of pelagic dives could be explained by dives in water depths beyond physiological dive limits or opportunistic prey encounters in midwater. The first explanation is unlikely, as the majority of all dives occurred in depths of less than 150 m of water and some seals dove to the benthos in water exceeding 300 m depth. The low relative probability of repeat pelagic dives observed in this study suggests grey seals are opportunistically encountering prey items *en route* to the benthos, whereas if prey were aggregated midwater we would expect successive pelagic dives. A number of studies have combined dive profiles with stomach temperature loggers [22], jaw accelerometers [23] and cameras [24] to provide a direct link between dive behaviour and feeding events. It would be an interesting avenue of research to combine these techniques with our analysis to determine if transitions from benthic to pelagic dives are actually associated with prey encounter or capture, to further explore this hypothesis.

Numerous studies have investigated relationships between diving marine mammals and habitat. Associations have been found with features such as continental shelf breaks [25], depth strata [26], sea-surface temperature [3], chlorophyll-a concentration and light attenuation [27], and ecoregions based on combinations of these [5]. However, these have been limited to broad-scale oceanographic features, and in some analyses, a generally low amount of variation explained by models suggests that diving behavior varies in response to finer-scale biological, temporal, and/or physical features [4]. Limited information exists on finer-scale habitat use with respect to sediment type [28], an important factor for demersal feeders such as the grey seal, as sediment type influences the distribution and abundance of available prey [16], [17], [18]. Harbour seals have been documented to forage in areas dominated by sandy sediment, resulting in a diet dominated by fish species associated with this habitat [21]. However, a direct link between individual dives and sediment could not be made, since only broad foraging areas were identified using radio-tagged individuals. Furthermore, it is unclear how these seals responded to changes in habitat encountered during successive dives.

Tagged grey seals in our study foraged over a range of habitats, from fine sediments such as mud/sand through to three-dimensionally complex rock. The frequency of pelagic dives decreased with increasing habitat complexity, demonstrating that dive behaviour changes in relation to habitat and the distinct prey associations that occur over contrasting sediments. Transition frequencies will likely depend on size of habitat blocks and the degrees of habitat fragmentation occurring within blocks, which is likely to vary geographically and by habitat type. While relatively fine, the resolution of the sediment data (167×333 m) was coarser than the location resolution, so may not fully account for highly heterogeneous or fragmented habitats within each mapped sediment type. However, the low probability of transitions between mapped sediment types (e.g. rock to fine sediment, mixed to fine sediment, etc.), suggests that seals were not foraging along the boundary between them.

These results are consistent with the known diet of grey seals. Grey seals are opportunistic, generalist feeders, consuming a wide range of prey species [11], [12], [13]. In Irish waters, diet consists mainly of demersal inshore species, with sandeels (*Ammodytes spp*.), dragonets (*Callionymus spp.*), and gadoids such as bib (*Trisopterus luscus*), poor cod (*Trisopterus minutus*), blue whiting (*Micromesistius poutassou*), pollock (*Pollachius pollachius*), and saithe (*Pollachius virens*) dominating [19], [29]. Large numbers of benthic species such as juvenile flatfish and pelagic schooling sandeels use sandy habitats [30], and the probability of pelagic dives over sandy habitats is consistent with foraging for sandeel shoals. Juvenile gadoids aggregate on the bottom by day, utilizing complex substrata such as rock and cobble [31]. An increased likelihood of dives to the seabed over rocky substrates is consistent with foraging on juvenile gadoids, with gadoids below minimum landing size being common prey of grey seals in Ireland [29]. Rocky habitats also provide a heterogeneous environment, potentially resulting in more diverse prey assemblages, and are less frequently disturbed by the fisheries industry than non-rocky substrates. Therefore, it is possible that grey seals make benthic dives more frequently in rocky habitats because more prey are available than in areas fished commercially. These results suggest potentially low direct resource competition with offshore fisheries, which is consistent with a finding of low spatial overlap between grey seals and the offshore fishery off the west coast of Ireland [32]. However, this study does not include operational interactions with commercial fisheries, where high levels of seal damage to catches occur at the nets (unpublished data).

The prevalence of pelagic dives at night suggests that light levels are influencing foraging behaviour, probably indirectly. Diurnal foraging patterns have been observed in other marine vertebrates [4], [33], [34], and are suggested to be a response to the diurnal migration of prey up into the water column at night, enabling seals to forage midwater.

Our ability to gather accurate location data has greatly enhanced our ability to investigate behavioural responses of key marine predators to fine-scale environmental factors. Even with additional error associated with interpolating dive locations between reliable position fixes, total error (error of original position fix plus additional error of interpolated points; ±87 m) is less than the spatial resolution of the sediment data used in this study (∼167×333 m). Fine-scale habitat data, and accurate locations enabled us to demonstrate habitat-mediated changes in dive behaviour across relatively broad habitat classifications. The proportion of pelagic dives decreased as seals foraged over increasingly coarser sediments from mud/sand through to rocky substrates. This approach could be further used to make more accurate predictions of habitat use in data-poor areas, and investigate contentious issues such as resource overlap and competition between top predators and human activities.

## Supporting Information

Figure S1 **Setting thresholds for dive state classification.** A) Plot of proportional error in calculations of proximity to the benthos. Proximity was calculated as dive depth/bathymetric depth. In shallow water, values above 1 (an easily identifiable error where dive depth exceeds chartered bathymetry) became more common. The plot shows the proportion of dives showing proximity>1, binned by five metre depth intervals. In water deeper than 50 m, this error disappears so we have more confidence in estimates of proximity to the benthos. B) The distribution of proximity to the benthos, showing a peak around 1. The threshold ratio for benthic (red) versus pelagic (blue) was set at 0.95, which was just before the point of inflection in the Cumulative Distribution Function shown in (C).
(TIFF)

## References

[1] Hooker SK, Biuw M, McConnell B, Miller PJO, Sparling CE (2007) Bio-logging science: logging and relaying physical and biological data using animal-attached tags. Deep Sea Res Part 2 Top Stud Oceanogr 54: 177–182.

[2] Wilson RP, Vandenabeele SP (2012) Technological innovation in archival tags used in seabird research. MEPS 451: 245.

[3] Bradshaw C, Higgins J, Michael K, Wotherspoon S, Hindell MA (2004) At-sea distribution of female southern elephant seals relative to variation in ocean surface properties. ICES J Mar Sci 61: 1014–1027.

[4] Burns J, Hindell MA, Bradshaw C, Costa DP (2008) Fine-scale habitat selection of crabeater seals as determined by diving behavior. Deep Sea Res Part 2 Top Stud Oceanogr 55: 500–514.

[5] Lea MA, Dubroca L (2003) Fine-scale linkages between the diving behaviour of Antarctic fur seals and oceanographic features in the Southern Indian Ocean. ICES J Mar Sci 60: 1–13.

[6] Simmons S, Crocker DE, Kudela R, Costa DP (2007) Linking foraging behaviour of the northern elephant seal with oceanography and bathymetry at mesoscales. MEPS 346: 265–275.

[7] Hays GC (2001) The implications of location accuracy for the interpretation of satellite-tracking data. Anim Behav 61: 1035–1040.

[8] Ryan P, Petersen S, Peters G, Grémillet D (2004) GPS tracking a marine predator: the effects of precision, resolution and sampling rate on foraging tracks of African penguins. Mar Biol 145: 215–223.

[9] Costa DP, Robinson PW, Arnould JPY, Harrison AL, Simmons SE, et al (2010) Accuracy of ARGOS locations of pinnipeds at-sea estimated using Fastloc GPS. PloS one 5: e8677. 20090942 10.1371/journal.pone.0008677 PMC2806907

[10] Hazel J (2009) Evaluation of fast-acquisition GPS in stationary tests and fine-scale tracking of green turtles. JEMBE 374: 58–68.

[11] Beck CA, Iverson SJ, Bowen W, Blanchard W (2007) Sex differences in grey seal diet reflect seasonal variation in foraging behaviour and reproductive expenditure: evidence from quantitative fatty acid signature analysis. J Anim Ecol 76: 490–502. 17439466 10.1111/j.1365-2656.2007.01215.x

[12] Bowen WD, Beck CA, Iverson SJ, Austin D, McMillan JI (2006) Linking predator foraging behaviour and diet with variability in continental shelf ecosystems: Grey seals of eastern Canada. In: Boyd IL, Wanless S, Camphuysen CJ, editors. Top Predators in Marine Ecosystems-Their Role in Monitoring and Management. Cambridge: Cambridge University Press, pp. 63–81.

[13] Ridoux V, Spitz J, Vincent C, Walton MJ (2007) Grey seal diet at the southern limit of its European distribution: combining dietary analyses and fatty acid profiles. JMBA 87: 255–264.

[14] McConnell BJ, Fedak MA, Lovell P, Hammond PS (1999) Movements and foraging areas of grey seals in the North Sea. J App Ecol 36: 573–590.

[15] Cronin M, Pomeroy P, Jessopp M (2013) Size and seasonal influences on the foraging range of female grey seals in the northeast Atlantic. Mar Biol 160: 531–539.

[16] Williams A, Bax NJ (2001) Delineating fish-habitat associations for spatially based management: an example from the south-eastern Australian continental shelf. Mar Freshw Res 52: 513–536.

[17] Anderson TJ, Syms C, Roberts DA, Howard DF (2009) Multi-scale fish–habitat associations and the use of habitat surrogates to predict the organisation and abundance of deep-water fish assemblages. JEMBE 379: 34–42.

[18] Chittaro P (2004) Fish-habitat associations across multiple spatial scales. Coral Reefs 23: 235–244.

[19] Brown SL, Bearhop S, Harrod C, McDonald RA (2012) A review of spatial and temporal variation in grey and common seal diet in the United Kingdom and Ireland. JMBA 92: 1711–1722.

[20] Cronin M (2011) The conservation of seals in Irish waters: How research informs policy. Marine Policy 35: 748–755.

[21] Tollit DJ, Black AD, Thompson PM, Mackay A, Corpe HM, et al (1998) Variations in harbour seal Phoca vitulina diet and dive-depths in relation to foraging habitat. J Zool 244: 209–222.

[22] Austin D, Bowen WD, McMillan JI, Boness DJ (2006) Stomach temperature telemetry reveals temporal patterns of foraging success in a free-ranging marine mammal. J Anim Ecol 75: 408–420. 16637994 10.1111/j.1365-2656.2006.01057.x

[23] Viviant M, Trites A, Rosen D, Monestiez P, Guinet C (2010) Prey capture attempts can be detected in Steller sea lions and other marine predators using accelerometers. Polar Biol 33: 713–719.

[24] Hooker SK, Boyd IL, Jessopp MJ, Cox O, Blackwell J, et al (2002) Monitoring the prey-field of marine predators: Combining digital imaging with datalogging tags. Mar Mamm Sci 18: 680–697.

[25] McConnell BJ, Chambers C, Fedak MA (2004) Foraging ecology of southern elephant seals in relation to the bathymetry and productivity of the Southern Ocean. Antarct Sci 4: 393–398.

[26] Arnould JPY, Kirkwood R (2007) Habitat selection by female Australian fur seals (*Arctocephalus pusillus doriferus*). Aquat Conserv 17: S53–S67.

[27] Jaud T, Dragon A, Garcia J, Guinet C (2012) Relationship between Chlorophyll a concentration, light attenuation and diving depth of the Southern Elephant Seal *Mirounga leonina* . PLoS ONE 7: e47444 doi:47410.41371/journal.pone.0047444. 23082166 10.1371/journal.pone.0047444 PMC3474817

[28] Parrish FA, Craig MP, Ragen TJ, Marshall GJ, Buhleier BM (2000) Identifying diurnal foraging habitat of endangered Hawaiian monk seals using a seal-mounted video camera. Mar Mamm Sci 16: 392–412.

[29] Gosch M (2010) Diet studies of the grey seal (*Halichoerus grypus*) off the south-west of Ireland. MSc thesis Cork, Ireland: University College Cork.

[30] Boelens RGV, Maloney DM, Parsons AP, Walsh AR (1999) Ireland's marine and coastal areas and adjacent seas. An environmental assessment. Dublin: Marine Institute.

[31] Kamenos NA, Moore PG, Hall-Spencer JM (2004) Small-scale distribution of juvenile gadoids in shallow inshore waters; what role does maerl play? ICES J Mar Sci 61: 422–429.

[32] Cronin M, Gerritsen H, Reid DG (2012) Evidence of low spatial overlap between grey seals and a specific whitefish fishery off the west coast of Ireland. Biol Conserv 150: 136–142.

[33] Boyd IL, Arnould JP, Barton T, Croxall JP (1994) Foraging behaviour of Antarctic fur seals during periods of contrasting prey abundance. J Anim Ecol 63: 703–713.

[34] Wilson RP, Pueta K, Bost CA, Culik BM, Bannasch R, et al (1993) Diel dive depth in penguins in relation to diel vertical migration of prey: whose dinner by candlelight? MEPS 94: 101–104.

