# Supplementary figures and images for: Habitat-Mediated Dive Behavior in Free-Ranging Grey Seals
Source: PLoS One. 2013 May 7;8(5):e63720. doi: 10.1371/journal.pone.0063720 (PMC3646810; doi:10.1371/journal.pone.0063720)

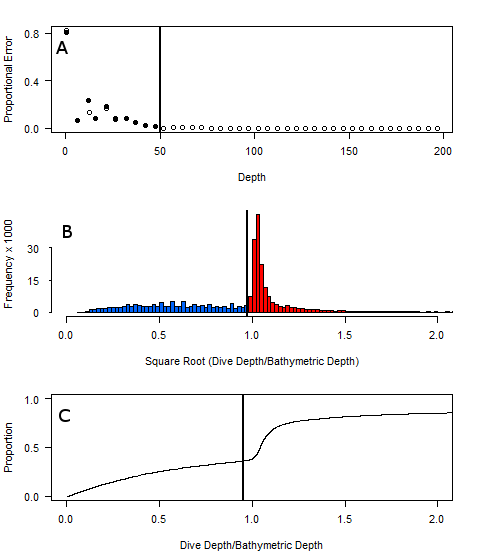

Supplement: Figure S1 — Setting thresholds for dive state classification. A) Plot of proportional error in calculations of proximity to the benthos. Proximity was calculated as dive depth/bathymetric depth. In shallow water, values above 1 (an easily identifiable error where dive depth exceeds chartered bathymetry) became more common. The plot shows the proportion of dives showing proximity>1, binned by five metre depth intervals. In water deeper than 50 m, this error disappears so we have more confidence in estimates of proximity to the benthos. B) The distribution of proximity to the benthos, showing a peak around 1. The threshold ratio for benthic (red) versus pelagic (blue) was set at 0.95, which was just before the point of inflection in the Cumulative Distribution Function shown in (C). (TIFF) [file pone.0063720.s001.tiff]
